# Text of the Correspondence

**Published:** 1916-11-18

**Authors:** 


### STATE REGISTRATION.

### THE CENTRAL COMMITTEE AND THE COLLEGE OF NURSING.

### TEXT OF THE CORRESPONDENCE.

### Letters from the Central Committee.

Central Committee for the State Registration of Nurses,
431 Oxford Street, London, W.,
July 17, 1916.

Dear Mr. Stanley,—I beg to enclose to you a copy of the second draft of your Bill, with the amendments interpolated as agreed by the Central Committee for the State Registration of Nurses at its meeting on Thursday, July 13.

The Committee was gratified to find that four clauses as adopted by it at a former meeting had been incorporated in the Bill. The Committee, however, felt that the draft before it on the 13th instant needed considerable amendment and extension.

**[Note.—These four clauses dealt respectively with**

**(a) Rules electing direct representatives of the nurses by ballot (now Clauses 4 (1) (i.) (b) in the fourth draft of the Bill, hereinafter called the Bill).**

**(b) Making provision for the admission to the Register of existing nurses (now Clause 6 of the Bill).**

**(c) Defining who may be registered at the expiration of the time of grace (now Clause 7 of the Bill).**

**(d) Providing for an appeal to a Court of Law by a registered nurse against a decision of the Council removing him, or her, from the Register (now Clause 15 of the Bill).]**

### The Central Committee's Remarks.

Clause 2 (3).

This clause provides that every nurse, whether certificated or not, during the time of grace, and every male and mental nurse, becomes *ipso facto* a member of the College. This is a point which requires further consideration if any prestige is to be given to membership of the College of Nursing.

**[Note.—The original clause thus criticised read as follows:—**

**2. (3) Every nurse registered under this Act shall be entitled to vote at elections of the Council, and without further fee to become a member of the College of Nursing. The force of the criticism was admitted, and in the Bill the clause now appears as follows:—**

**2. (3) Every nurse registered under this Act shall be entitled to vote at elections of the Council, and '*if registered on the General Register,* without further fee, to become a member of the College of Nursing."]**

Clause 4 (1) (i.).

Whilst agreeing to the inclusion on the governing body of representatives of the nurse-training schools, the nurse representatives regret the necessity for the inclusion of such a principle.

**[Note.—The paragraph to which this refers relates to rules to be made under the Act, and now runs as follows:—**

**4. 1. (i.) Regulating the constitution and proceedings and the mode of election and of retirement of the Council and providing for the representation thereon of the Privy Council and any Government Department, of the nurse-training schools, of the medical profession, and of the nurses on the Supplementary Register.**

**The clause as thus altered accepts the suggestion of the Central Committee to delete the words "if thought fit," thus laying it down unconditionally that repre-**

**STATE REGISTRATION**—(*continued*).

**sentation should be given on the permanent Council to the various bodies enumerated.]**

SUGGESTED NEW SUB-SECTIONS.

A new paragraph would, if adopted, prevent the disorganisation of an annual election and materially decrease the cost of conducting the business of the Council.

**[Note.—The new sub-sections suggested, as stated in the Memorandum, are as follows:—**

**"At the expiration of two years from the date of the first constitution of the Council, and at the expiration of every subsequent period of three years, the members of the Council, whether elected or appointed, shall retire, but a retiring member shall be eligible for re-election or re-appointment."**

**This clause was not adopted in the Bill, but the question as to how the Council should retire was left to be defined later by the rules to be made under the Act (see Clause 4 (1) (i.) above).]**

Dr. Chapple specially pressed for the two new sub-sections (ix. and x.) in Clause 4.

**[Note.—These sub-sections are as follows:—**

**(ix.) Providing for a register of hospitals qualified to give a course of training prescribed by the Council.**

**(x.) Providing for the appointment of visitors to such training schools, and for the addition to or removal from the list of such training schools.**

**These clauses seemed to be unnecessary in view of Clause 7 in the Bill requiring the training to be had "under a definite curriculum prescribed by the Council in a nurse-training school, or schools, recognised by the Council," and the inclusion of the latter clause would scarcely facilitate the passage of a Bill which it was desired to make non-contentious.]**

CLAUSE 5.

The Committee considers that the Nurses Act, like the Medical and Midwives Acts, should include the names of the authorities which are to nominate their governing body—the General Nursing Council. The nurses feel most strongly on this point, and under Clause 4 they object to the words "if thought fit" in Section 1 (i.), as they recognise that if inserted there is no security whatever for their direct representation on their own governing body.

**[Note.—This clause as prepared by the Central Committee runs as follows:—**

**"On the passing of this Act and for a term of two years the Council shall consist of forty-five persons, of whom two shall be appointed by the Privy Council, three by Local Government Boards, three by the Council of the British Medical Association, one by the Medico-Psychological Association, six by the nurse-training schools, fifteen by the Central Committee for the State Registration of Nurses, fifteen by the College of Nursing, Ltd."**

**As it appears in the College Bill the clause is as follows:—**

**5. (1) On the passing of this Act and for a term of two years the Council shall consist of the following forty-five persons so long as they may be willing to act:—**

**(2) Casual vacancies may be filled up by co-optation.**

**The reasons why the College has adhered to this clause are set out in Mr. Stanley's letter of October 4 (see next page). The further point is conceded, and "if thought fit" no longer appears in Clause 4 (1) (i.) of the Bill.]**

NEW CLAUSES 6 AND 7.

Dr. Chapple and the Committee also press for the insertion of new Clauses 6 and 7, defining the "provision for existing nurses" and "who may be registered," and Dr. Goodall understood that you had agreed to the principle of recognising a fever nurse's qualification, in addition to general experience, on the general register, and he expressed the opinion that it would cause unnecessary friction and trouble if this provision is not made in the Act.

**[Note.—As already stated, Clauses 6 and 7 to this effect have been included.]**

NEW CLAUSE 14.

The privilege of appeal by a hospital if aggrieved by the refusal of the Council to recognise it as a training school was insisted upon by the House of Lords when the Nurses' Registration Bill was passed in 1908.

**[Note.—Not accepted. It is difficult to see how any Court of Law can be as competent to decide upon the claims of a nurse-training school to be recognised as the General Nursing Council, consisting, as it must do, largely of experts in nursing questions. At any rate, if such a clause is to be included in the Bill it should be left to Parliament to insist on it.]**

RE DEFINITION CLAUSE.

Dr. Chapple strongly urged that the definition clause of what is a registered nurse and a registered male and mental nurse should be inserted.

**[Note.—The Memorandum sets out this clause as follows:—**

**In this Act the term 'registered nurse' means a woman nurse who is for the time being registered in the woman nurses' general register, the term 'registered male nurse' means a male nurse who is for the time being registered in the male nurses' supplementary register. The term 'registered mental nurse' means a mental nurse who is for the time being registered in the mental nurses' supplementary register.**

**The above clause seems unobjectionable, but not necessary, and has not, therefore, been included in the Bill.]**

If you wish to see Dr. Goodall and myself again on any of these points we shall be pleased to attend.

It was understood that the memorandum and articles of association of the College of Nursing, Limited, are at present under the consideration of its Council, so their discussion was deferred.—I am, yours very truly,

(Signed) ETHEL G. FENWICK,
Nurse Hon. Secretary.

To the Honble. Arthur Stanley, M.P., M.V.O., C.B.

---

Central Committee for the State Registration of Nurses,
431 Oxford Street, London, W.
September 30, 1916.

DEAR SIR,—At a full meeting of the Central Committee for the State Registration of Nurses, which was held on the 28th inst., the fourth draft of the Nurses' Registration Bill, as approved by the Council of the College of Nursing, Ltd., dated July 27, 1916, was considered by the Committee.

The Committee were agreed—

"That, without prejudice to the further consideration of other amendments, the Council of the College of Nursing be informed that this Committee consider the adoption of the following amendments to the Bill drafted by the College to be so essential that, unless they are accepted by the College, the Central Committee cannot continue the negotiations."

The amendments considered essential are numbered Clause 5 (1), Clause 6, and Clause 7 in its entirety, as agreed by the Central Committee on July 13 last, and forwarded on July 17 for the consideration of your Council.

And, as agreed by the Central Committee on September 28 last, such a modification of Clause 4, sub-section (1), as shall secure the following composition of the permanent Council :—

That two-thirds of the permanent Council shall be elected by the registered nurses and one-third as defined in the constitution of the preliminary Council, as follows :

Two persons appointed by the Privy Council;

Three by the Local Government Boards (one appointed by the Local Government Board of England, Scotland, and Ireland respectively);

Three by the Council of the British Medical Association;

One by the Medico-Psychological Association; and

Six by the nurse-training schools.

We are further instructed by the Central Committee to inform you that, at the same meeting, the Executive Committee were directed to consider their own Nurses' Bill of 1910, to make such amendments as they deemed necessary, and to be prepared to present it to Parliament in the event of failure of agreement between the Central Committee and the College of Nursing, Ltd.

Awaiting a reply at the earliest possible date, we are, dear Sir, yours faithfully,

(Signed) ETHEL G. FENWICK,
Hon. Nurse Secretary.
(Signed) E. W. GOODALL,
Hon. Medical Secretary.

To the Hon. Arthur Stanley, M.P., M.V.O., C.B.,
Chairman of the Council,
College of Nursing, Ltd.

### The Hon. Arthur Stanley's Reply.

The College of Nursing, Limited,
83 Pall Mall, London, S.W.
October 4, 1916.

DEAR MADAM,—As your Committee are desirous of receiving an answer to their letter of the 30th ultimo at the earliest possible date I am taking upon myself to reply without having had an opportunity of consulting the Council of the College of Nursing, with which, of course, the ultimate decision lies, and I write with the greater readiness as I cannot but feel that the differences between us are matters rather of expediency and policy than of fundamental principles. Both parties are out for State registration; it is merely a question how to get it with a minimum of friction and delay.

To consider the amendments of the Central Committee to the fourth draft of the Council's Bill *seriatim* :—

CLAUSE 5 (1).

The difference here is in the main a difference as to the mode of appointment of the first Council. The Central Committee's Bill excludes names and suggests a list of appointing bodies; the Council's Bill proposes to set out the actual names of the members of the first General Nursing Council. It is a question which method is more likely to conciliate opposition and facilitate the passing of the Bill.

Against the clause as proposed by your Committee the following criticisms at once suggest themselves :

1. No representation of Poor-Law authorities or Poor-Law nurses.
2. No representation of Scotland or Ireland.
3. The difficulty of choosing six out of the whole of the nurse-training schools of the country without exciting jealousy amongst the excluded.
4. The cast-iron rigidity of the proposal and the certainty that in Parliament objections would be raised which might either wreck the Bill or result in a Council of quite unwieldy dimensions.

On behalf of the Council's proposal to set out the names of the first General Nursing Council I urge :—

1. That it is more difficult in Parliament to object to the name of an individual than to a list of nominating bodies.
2. That members are more likely to view favourably a Bill for registration if they know of whom the first General Council is actually to consist than if they are asked to entrust the nomination of it to bodies of whom they know little or nothing.

Here, of course, I ought perhaps to repeat that there is no intention on my part of receding from the position I have taken up all along, that the Central Committee, which has done so much for State registration of nurses, should have just as many nominees on the first Council as the College has, and I should be very glad if the Central Committee would empower certain of its members in whom it has confidence to enter into informal consultation with myself and a few members of the Council of the College, so as to start right away upon settling a provisional list for the first Council. I fancy that if we once got to work round a table many of the difficulties anticipated would be found practically to solve themselves, and that we could get out a Council which would command the confidence of the profession, of the public, and of the Houses of Parliament.

CLAUSE 6.

There are two differences between the clause as drafted by the Council of the College and the Central Committee respectively. The Council says "within three years," the Central Committee "within two years."

As a matter of fact the Council had originally put "two years," but it was pointed out that a probationer enters her training school normally for a three years' term of service, with certain regulations as to examinations and curriculum, which will very likely be disturbed when the Registration Bill becomes law. It would be unfair to bring her under these new regulations in the course of a training begun under other conditions, and hence the three years' grace to date from the passing of the Act.

The other difference amounts to this, that under the clause as drafted by the Central Committee all registration of existing nurses would be stopped as soon as ever the Bill passed until the first Council had made rules under Clause 4 (iv.) "regulating the admission to the Register of persons already in practice as trained nurses at the passing of this Act." The clause as drafted by the Council merely keeps the work of registration continuing on the old lines until modified by rules made under the Act.

As all "rules" have to be approved by the Privy Council and laid before Parliament, some delay is pretty sure to occur, and thus the Register of Nurses, unless registration goes on automatically in the interval, will be less complete than it need be when the nurses elect the permanent Council.

CLAUSE 7.

The difference here seems to lie in the use by the Council of the words "nurse-training school or schools" rather than the words "hospital or hospitals." The

**STATE REGISTRATION**—(*continued*).

former were approved because the Bill deals with hospitals as places of education, as "training schools" in fact. Further, distinctions are often made between hospitals and Poor-Law infirmaries. Moreover, the custom of the North Country speaks of infirmaries rather than hospitals, and finally the clause as drafted by the Central Committee, requiring a nurse to have had a training under a definite curriculum prescribed by the Council in the *wards* of a hospital, would, strictly interpreted, exclude from the curriculum the time put in by her in the casualty or out-patient department, which is clearly not meant.

Clause 4 (i.).

Here again, as in Clause 5 (i.), the question is largely one of expediency. The Council of the College has been very strongly advised by their solicitors and Parliamentary counsel to lighten the Bill as much as possible by confining its provisions to main principles rather than to elaborating details, and the main principle we are interested in is that the nurses should appoint two-thirds of the permanent Council, and this, as you are aware, is laid down definitely in 4 (i.) (*a*).

The proportionate representation of their interests may very well be left, I think, to the first Council; the attempt to define how it should be done in the Bill itself would almost certainly lead to trouble, and might even be fatal to the measure.

Perhaps I ought to say, in conclusion, that everyone of Parliamentary experience must be aware that there is absolutely no hope of passing a contentious Bill in the present state of public business, and that, therefore, an agreed measure is the only chance we have of obtaining the State recognition of the nursing profession. Even then it is doubtful whether the opposition still to be expected from quarters which have been hostile hitherto may not be powerful enough to defeat our combined efforts.

I hope that the explanations that I have given of our Bill may convince your Committee that, as I said at the beginning of this letter, our differences concern details, not fundamentals, and consequently ought not to be incapable of mutual adjustment.—Yours sincerely,

(Signed) A. Stanley.

Mrs. Bedford Fenwick.

### Further Correspondence.

Central Committee for the State Registration of Nurses,
431 Oxford Street, London, W.
October 7, 1916.

Dear Mr. Stanley,—I beg to thank you for your personal letter of explanation dated October 4. Having consulted Dr. Goodall, the Hon. Medical Secretary, we desire to inform you that we have no further jurisdiction in the matter under discussion.

The Central Committee for the State Registration of Nurses directed us to forward to you, as Chairman of the Council of the College of Nursing, Ltd., the result of its deliberations at its meeting on September 28 last, and to ask that an official reply might be sent at your earliest convenience.

Awaiting this reply, I remain, yours faithfully,

(Signed) Ethel G. Fenwick,
Hon. Nurse Secretary.

To the Honble. Arthur Stanley, M.P., Chairman of the Council of the College of Nursing, Ltd.

---

The College of Nursing, Ltd.,
83 Pall Mall, London, S.W.,
October 10, 1916.

Dear Madam,—Your letter of the 7th instant only reached me this morning. In reply I have to say that I submitted my letter to you of October 4 to my Council at their meeting on October 5, and it was approved. Will you, therefore, take my letter of October 4 as my official answer to your letter of September 30?—I am, dear Madam, yours faithfully, (Signed) Arthur Stanley.

To Mrs. Bedford Fenwick.

---

Central Committee for the State Registration of Nurses,
431 Oxford Street, London, W., October 16, 1916.

Dear Sir,—I beg to acknowledge your letter of the 10th inst., and note that your former letter, dated October 4, is the official answer for my Committee from the Council of the College of Nursing, Ltd.—I am, dear Sir, yours faithfully, (Signed) Ethel G. Fenwick,
Hon. Nurse Secretary.

To the Hon. Arthur Stanley, M.P., M.V.O., C.B., Chairman of the Council of the College of Nursing, Ltd.

Central Committee for the State Registration of Nurses,
431 Oxford Street, London, W., October 25, 1916.

Dear Sir,—We are directed to inform you that at the meeting of the Central Committee for the State Registration of Nurses, held in London on Saturday, October 21, your letter of October 4 was read and considered, and it was resolved :—

"That the Central Committee regrets it cannot recede from the position it has taken up and fully communicated to Mr. Stanley in the letter of September 30, and it has therefore determined to proceed with its own Bill."—We are, dear Sir, yours faithfully,

(Signed) Ethel G. Fenwick,
Hon. Nurse Secretary.
(Signed) E. W. Goodall,
Hon. Medical Secretary.

To the Honble. Arthur Stanley, M.P., M.V.O., C.B., Chairman of the Council of the College of Nursing, Ltd.

---

The College of Nursing, Ltd.,
6 Vere Street, Cavendish Square, London, W.
October 28, 1916.

The Hon. Nurse Secretary,
Central Committee for the State Registration of Nurses.

Dear Madam,—The Hon. Arthur Stanley wishes me to thank you for your letter of the 25th inst., and to say that it will be placed before the Council of the College at its next meeting.—I remain, yours truly,

(Signed) M. S. Rundle, Secretary.

---

The College of Nursing, Ltd.,
83 Pall Mall, S.W., November 7, 1916.

Dear Madam,—I am desired by the Council to acknowledge the resolution of the Central Committee for the State Registration of Nurses conveyed in your letter to me of the 25th ultimo, and to say that they much regret the abrupt termination by your Committee to negotiations for an agreed Bill at a moment when substantial progress was believed to have been made. They fear that the action so taken may result in still further postponing State recognition of the nursing profession.—I am, dear Madam, yours faithfully, (Signed) Arthur Stanley.

Mrs. Bedford Fenwick.

